# Role of neuropeptide FF in central cardiovascular and neuroendocrine regulation

**DOI:** 10.3389/fendo.2013.00008

**Published:** 2013-02-07

**Authors:** Jack H. Jhamandas, Valeri Goncharuk

**Affiliations:** ^1^Division of Neurology, Department of Medicine, Centre for Neuroscience, University of AlbertaEdmonton, AB, Canada; ^2^Russian Cardiology Research CenterMoscow, Russia

**Keywords:** RFamide, FMRFamide, NPFF1, NPFF2, hypothalamus, paraventricular nucleus, blood pressure, hypertension

## Abstract

Neuropeptide FF (NPFF) is an octapeptide belonging to the RFamide family of peptides that have been implicated in a wide variety of physiological functions in the brain including central cardiovascular and neuroendocrine regulation. The effects of these peptides are mediated via NPFF1 and NPFF2 receptors that are abundantly expressed in the rat and human brain. Herein, we review evidence for the role of NPFF in central regulation of blood pressure particularly within the brainstem and the hypothalamic paraventricular nucleus (PVN). At a cellular level, NPFF demonstrates distinct responses in magnocellular and parvocellular neurons of the PVN, which regulate the secretion of neurohypophyseal hormones and sympathetic outflow, respectively. Finally, the presence of NPFF system in the human brain and its alterations within the hypertensive brain are discussed.

## INTRODUCTION

An understanding of the mechanisms that regulate arterial blood pressure under physiological conditions and in the context of pathophysiological situations such as hypertension represents a major challenge. Essential hypertension is the most common form of hypertension in humans, although its cause is poorly understood. However, there is substantial evidence to indicate that essential hypertension may be related to elevated levels of sympathetic nervous activity, which originate within the central nervous system (CNS). Within the CNS, neural networks governing arterial blood pressure are contained within topographically segregated but interactive cell groups represented at all levels of the neuraxis. Amongst the many neurotransmitters and neuropeptides present in these autonomic regions, emerging evidence indicates that a group of RFamide peptides play an important role in CNS regulation of cardiovascular function.

## WHAT ARE RFamide PEPTIDES?

Historically, the cardioexcitatory peptide FMRFamide from the bivalve mollusc *Macrocallista nimbosa* was the first peptide isolated and identified with an Arg-Phe-amide C-terminus (Price and Greenberg, 1977). Since then many bioactive peptides have been isolated from invertebrates and vertebrates, and the extended family of peptides terminating in a penultimate Arg and an amidated Phe residue at the C-terminus (RFamide) exists in all phyla (Yang et al., 1985). These peptides are designated as FMRFamide related peptides (FaRPs) and collectively referred to as RFamide peptides. RFamide peptides have been identified to have diverse biological functions that include pain modulation, inhibition of food intake, regulation of water balance, and potent cardiovascular actions that are mediated through the peripheral and CNSs (Raffa, 1988; Murase et al., 1996; Panula et al., 1996; Hinuma et al., 2000; Zajac and Gouarderes, 2000; Sunter et al., 2001; Fukusumi et al., 2003; Samson et al., 2003; Dockray, 2004).

**FIGURE 1:**
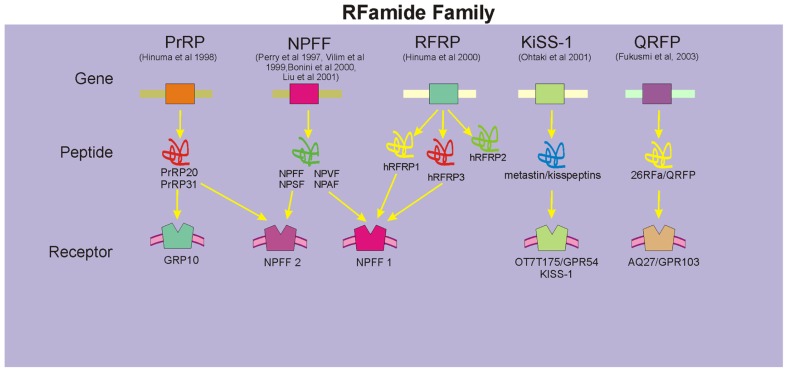
**Members of the mammalian RFamide family**. These include the prolactin releasing peptide (PrRP) family, the family of NPFF (and related peptides NPAF, NPSF, and NPVF), human RFamide related peptides (hRFRPs), metastin/kisspeptins, and QRFP family. Specific receptors for each family of peptides have been identified, although in a number of instances cross-talk amongst these peptides and their receptors exists (denoted by arrows).

The recent rapid accumulation of cDNA and genomic DNA sequence data and the development of bioinformatics have had a profound impact on the field of RFamide peptide research, especially on gene identification and analyses of RFamide peptides and their receptors. While some confusion exists on the precise nomenclature used in the literature, five genes encoding five prepropeptide precursors that yield five groups of RFamide peptides have been described in mammals (**Figure 1**). These include the prolactin-releasing peptide (PrRP) family (Hinuma et al., 1998), the family of neuropeptide FF (NPFF; and related peptides neuropeptide AF (NPAF), neuropeptide SF (NPSF), and neuropeptide VF (NPVF); Perry et al., 1997; Vilim et al., 1999; Bonini et al., 2000; Liu et al., 2001), human RFamide related peptides (hRFRPs; Hinuma et al., 2000; Fukusumi et al., 2001), metastin/kisspeptins (Ohtaki et al., 2001), and pyroglutamylated RFamide peptide (QRFP) (26RFa) family (Chartrel et al., 2003; Fukusumi et al., 2003). Of these, NPFF peptides and PrRP have been identified by our laboratory and others to play an important role in CNS regulation of cardiovascular function (Thiemermann et al., 1991; Allard et al., 1995; Jhamandas et al., 1998; Samson et al., 2000; Jhamandas and MacTavish, 2002). hRFRPs, which are encoded by a human gene, have a significant homology to the NPFF family of peptides and their receptors (Fukusumi et al., 2006). Members of the RFRP family (RFRP-1 and RFRP-3) have been recently identified as mammalian orthologs of the avian gonadotropin inhibitory hormone and administration of the selective NPFF receptor antagonist results in potent secretion of gonadotropins that is presumed to be mediated via the NPFF1 receptor (Pineda et al., 2010), Metastatin/kisspeptins have been shown to have anti-migratory effects *in vitro*, metastasis-inhibiting effects *in vivo* (Muir et al., 2001; Ohtaki et al., 2001), and identified to play an important role in regulation of puberty and reproduction via gonadotropin release (Richard et al., 2009). QRFPs are the most recently discovered members of the RFamide family and postulated to play a role in food intake and increased locomotor activity (Chartrel et al., 2003; Fukusumi et al., 2006; Bruzzone et al., 2007).

## NEUROPEPTIDE FF

### CHARACTERISTICS AND TISSUE DISTRIBUTION

Neuropeptide FF (Phe-Leu-Phe-Gln-Pro-Gln-Arg-Phe-NH_2_) is an important member of the RFamide peptide family that is present in the CNS and in the periphery of several mammalian species including humans (for review see Panula et al., 1996). Initial interest in NPFF stemmed from its ability to modulate the antinociceptive effects of opioids (Yang et al., 1985; Chen et al., 2006; Mouledous et al., 2010), however, emerging studies have shown that the neuropeptide may play an equally important role in the central processing of visceral autonomic signals related to feeding, generation of central cardiovascular responses, stress, and neuroendocrine regulation (Panula et al., 1996; Jhamandas and MacTavish, 2003; Simonin et al., 2006). NPFF was the first RFamide peptide to be identified in mammals (Yang et al., 1985). The gene for NPFF has been cloned from human, bovine, rat, and mouse tissue and is highly conserved amongst these species (Yang et al., 1985; Vilim et al., 1999; Hinuma et al., 2000). The precursor mRNA encodes for NPFF and other related peptides (NPAF, NPSF, and NPVF) and distribution of the NPFF mRNA in the brain matches that of NPFF immunoreactivity (Kivipelto et al., 1989; Vilim et al., 1999; Liu et al., 2001). Immunocytochemical and receptor autoradiographic studies reveal that brain regions involved in pain transmission, autonomic and endocrine regulation are enriched in NPFF and its binding sites (Kivipelto et al., 1989; Allard et al., 1992). Concentrations of NPFF and its receptors in the hypothalamus are amongst the highest in the brain (Bonini et al., 2000; Zajac and Gouarderes, 2000; Liu et al., 2001).

### LIGANDS AND RECEPTORS

Two NPFF receptors, NPFF1 (also referred to as OT7TO22) and NPFF2 (also known as HLWAR77), have been cloned and characterized (Bonini et al., 2000; Elshourbagy et al., 2000; Zajac and Gouarderes, 2000). Both receptors are G_i/o_ protein-coupled when expressed in Chinese hamster ovary cells or human embryonic kidney 293 cells (Kotani et al., 2001). These receptors demonstrate some of the highest levels of expression within the rat and human brain and spinal cord but data on distribution of specific NPFF receptor subtypes in these regions is controversial (Bonini et al., 2000; Zajac and Gouarderes, 2000). The emerging picture, based on autoradiographic binding, immunohistochemical and *in situ* hybridization studies, is that NPFF1 receptors are predominantly localized in the hypothalamus and the forebrain, whereas NPFF2 receptors are mainly within the spinal cord, the brainstem visceral autonomic sensory nuclei, and the hypothalamus (Bonini et al., 2000; Gouarderes et al., 2002; Zeng et al., 2003; Goncharuk and Jhamandas, 2004).

NPFF1 receptor has a high affinity for the avian peptide LPLRFamide and is the candidate receptor for NPVF, NPSF as well as hRPRF1 and hRPRF2 peptides (Bonini et al., 2000; Elshourbagy et al., 2000; Liu et al., 2001). NPFF2, on the other hand, binds NPFF and NPAF (Liu et al., 2001; Fukusumi et al., 2006). Interestingly, PrRP, for which GPR10 has been identified as the endogenous receptor (**Figure 1**), has a relatively high affinity for the NPFF2 receptor and may even have a higher efficacy at the NPFF2 receptor than NPFF (Engström et al., 2003). When administered intracerebroventricular (icv), PrRP has been observed to produce elevations in arterial blood pressure and heart rate that are strikingly similar to those evoked by icv NPFF and in fact can be blocked with the selective NPFF antagonist, RF9 (Ma et al., 2009). Thus, many of the postulated physiological functions of the newer members of the RFamide family, PrRP and hRFRPs, may in fact be mediated via NPFF1 and NPFF2 receptors.

In the past few years, with the synthesis of peptide analogs of NPFF and related peptides, the essential requirements for ligand recognition at the NPFF receptor have emerged (Vyas et al., 2006). However, a major impediment to delineating a physiological role for NPFF peptides has been a lack of suitable antagonists that show selectivity for each of the receptors. DesaminoYLFQPQRa was the first analog to attenuate morphine abstinence signs induced by NPFF (Malin et al., 1995; Prokai et al., 2001) but suffers from poor bioavailability and/or low affinity for NPFF receptors (Fang et al., 2005). PFR(Tic)amide has been shown to demonstrate antagonist activity toward NPFF effect *in vitro*, but behaves as an agonist *in vivo* (Chen et al., 2006). Neuropeptide Y (NPY) ligands such as BIBP 3226 have been reported to interact with NPFF receptors, likely on the basis of structural similarities between these receptors and the C-terminal end of NPY peptides (Mollereau et al., 2002; Fang et al., 2006). Unfortunately, BIBP 3226 and its derivatives that were most potent at the NPFF1 receptor were also able to displace NPY Y_1_ binding (Fang et al., 2005). The discovery of RF9, a *selective* antagonist at the NPFF receptor (Simonin et al., 2006) has represented a significant advance in dissecting the role of NPFF in a variety of physiological functions. This compound potently and selectively binds to NPFF receptors and indeed blocks the acute cardiovascular effects induced by icv NPFF. In addition, its chronic administration blocks delayed and long-lasting opioid-induced hyperalgesia.

## NPFF AND CENTRAL CARDIOVASCULAR AND NEUROENDOCRINE REGULATION

Experimental evidence supporting a key role for NPFF in cardiovascular regulation first became apparent in the mid-1980s when Roth et al. (1987) reported that two NPFF analogs could produce significant pressor effects when administered systemically. Subsequently, focal injections of NPFF into the brainstem nucleus of tractus solitarius, which is the first terminus for cardiovascular inputs originating from the periphery, resulted in an increase in blood pressure and bradycardia that could be attenuated with adrenergic antagonists (Laguzzi et al., 1996). NPFF-synthesizing neurons in the same brainstem nucleus were shown to be activated in response to hemorrhage and to a lesser extent acute drug-induced hypertension (Jhamandas et al., 1998). These NPFF neurons in turn project to more rostral brainstem and hypothalamic cardiovascular centers. Intrathecal and icv administration of NPFF has been demonstrated to evoke dose-dependent elevations in arterial blood pressure and heart rate (Jhamandas and MacTavish, 2002, 2003; Fang et al., 2010). Identity of neural circuits that participate in centrally generated NPFF responses have been best studied in the hypothalamus, where icv NPFF evokes activation of specific sets of chemically defined paraventricular nucleus (PVN) neurons, that control CNS humoral and autonomic outflow to the periphery (Jhamandas and MacTavish, 2003).

### WHOLE ANIMAL OBSERVATIONS

The PVN is viewed as a key site for homeostasis and a model nucleus for understanding the central regulation of autonomic and neuroendocrine function in the brain (Cunningham and Sawchenko, 1991). The magnocellular neurosecretory cells of the PVN synthesize either vasopressin or oxytocin and following stimulation, release these hormones from their axonal projections to the posterior pituitary into the systemic circulation (Sawchenko and Swanson, 1982). On the other hand, the parvocellular component of the PVN is more complex and consists of two broad categories of cells, neurosecretory cells and non-neurosecretory (autonomic) cells. The neurosecretory parvocellular neurons are located within the dorsal medial and periventricular PVN and their axons terminate on median eminence portal capillaries to facilitate the release of “factors” regulating anterior pituitary secretion. Neurons of this type for example express corticotrophin-releasing hormone or thyrotrophin-releasing hormone. Parvocellular non-neurosecretory (autonomic) neurons are located within the dorsal cap and ventral medial PVN and project their axons to the brainstem and the spinal cord. Some of the chemical messengers expressed in these types of cells include tyrosine hydroxylase, oxytocin, and somatostatin (Roland and Sawchenko, 1993; Dawson et al., 1998). Central administration of NPFF results in a preferential activation of oxytocin-synthesizing parvocellular PVN neurons that project to the brainstem. Oxytocinergic projections to the solitary-vagal complex have previously been shown to modulate baroreflex control of heart rate and other aspects of circulatory control (Higa et al., 2002; Vela et al., 2010). On the other hand, icv NPFF does not activate the magnocellular vasopressin-secreting PVN neurons as measured by Fos immunohistochemistry suggesting that the effects of NPFF on these subset of PVN neurons are inhibitory (Jhamandas et al., 2006). The latter posit being supported by observation from electrophysiological studies where NPFF inhibits activity of magnocellular vasopressin neurons (see below).

### CELLULAR ACTIONS OF NPFF

**FIGURE 2:**
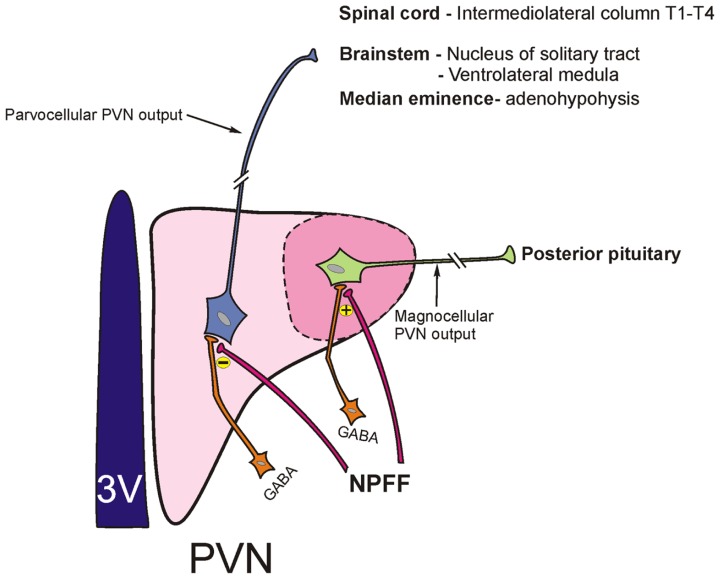
**Schematic depicting parvocellular and magnocellular components of the hypothalamic paraventricular nucleus (PVN) and their projection sites**. NPFF differentially modulates GABAergic input (originating from the adjacent subPVN region) to parvocellular and magnocellular neurons of the PVN. NPFF inhibits activity of GABAergic terminals that project to the parvocellular PVN neurons resulting in a disinhibition of these neurons. On the other hand, NPFF augments GABA synaptic input to magnocellular PVN neurons.

Work from whole animal experiments described above suggests that the differential activation of subsets of hypothalamic PVN neurons may result from distinct effects of NPFF on synaptic activation of magno- and parvocellular neurons of this nucleus. Indeed, cellular electrophysiological recordings from hypothalamic brain slices reveal that NPFF increases the inhibitory synaptic drive to magnocellular PVN neurons through a GABA-synthesizing network of interneurons located within the subPVN region (Roland and Sawchenko, 1993; Jhamandas et al., 2006; **Figure 2**). This observation of NPFF augmenting an inhibition of magnocellular vasopressin-secreting PVN cells fits well with the *in vivo* hormone release data, which shows that hypovolemia-induced vasopressin release from the pituitary is blunted by centrally administered NPFF (Arima et al., 1996). On the other hand, NPFF presynaptically *disinhibits* the GABAergic input to the parvocellular PVN, thereby increasing the net excitability of these neurons (**Figure 2**). NPFF also exerts a distinct depolarizing [tetrodotoxin (TTX)-independent] postsynaptic effect on parvocellular PVN neurons (Jhamandas et al., 2007). NPFF-induced excitation of parvocellular PVN neurons would be expected to result in an increase in autonomic outflow and sympathetic activation which is precisely what is observed with acute infusions of icv NPFF in the conscious animal. At present the lack of selective antagonists acting at NPFF1 versus NPFF2 receptors makes it difficult to determine whether the differential effects of NPFF are in fact mediated via distinct NPFF receptor subtypes. However, PrRP, which binds to NPFF2 receptors, shows a similar profile of electrophysiological effects on parvocellular PVN neurons as NPFF. Moreover RF9, a selective NPFF receptor antagonist, blocks PrRP actions on these neurons suggesting that both the PrRP and the NPFF effects in the parvocellular PVN are likely NPFF2 receptor mediated (Ma et al., 2009).

### NPFF AND HUMAN HYPERTENSION

There is currently a paucity of knowledge on the role of NPFF, and RFamide peptides in general, in human pathophysiological states such as essential hypertension. All of our current knowledge on the role of NPFF in the regulation of arterial blood pressure is derived from paradigms that rely on studying acute effects of this peptide in experimental animal models, which do not recapitulate the chronic human hypertensive condition. Nonetheless, anatomical relationships between NPFF and its receptors within autonomic centers in the human brain may provide important clues as to the role of this peptide in diverse biological functions. In this regard, immunohistochemical data from our laboratory over several years has identified striking similarities in the distribution of NPFF and its receptors, NPFF1 and NPFF2, in the normal human brain compared to the rat, a species in which much of the behavioral and physiological studies have been done to date (Goncharuk and Jhamandas, 2004, 2008; Goncharuk et al., 2006). In these studies, we have identified significant numbers of NPFF fibers, NPFF1, and NPFF2 receptors in the human parvocellular PVN. The relative preponderance of NPFF (and its receptors) and its intimate anatomical relationship to important cardiovascular regulatory peptides such as corticotropin releasing hormone (CRH) in human hypothalamus suggests an important role for this peptide in hypertension. Interestingly, an up-regulation of CRH-secreting cells in the human hypothalamic PVN of patients who suffered from essential hypertension has been reported (Goncharuk et al., 2001, 2002). Recent immunohistochemical observations from post-mortem brain tissue of hypertensive individuals and age-matched controls indicate a marked reduction of NPFF in discrete cardiovascular brainstem and hypothalamic nuclei of hypertensives (Goncharuk et al., 2011, 2012). In these studies, NPFF immunoreactivity was severely reduced in a subnuclear zone adjacent to the hypothalamic PVN and supraoptic nucleus, a site where dense networks of GABAergic neurons reside. These GABAergic neurons have been identified to mediate arterial baroreceptor inputs that control the release of the pressor hormone vasopressin from the neurohypophysis (Jhamandas et al., 1989). Thus loss of NPFF input to GABaergic cells has the potential to dysregulate cardiovascular reflexes and control of arterial blood pressure.

## CONCLUSION

Emerging evidence indicates that structure of RFamide peptides including NPFF is remarkably conserved during evolution. What makes these peptides attractive as therapeutic targets is that they are involved in essential functions such as pain, appetite and feeding, stress, and cardiovascular regulation. Anatomical, molecular, and physiological studies indicate that NPFF plays an important role in brain control of neurohormones and sympathetic outflow. Advances in identification and pharmacology of NPFF receptors and the availability of new and specific antagonists such as RF9 provide a unique opportunity to identify the specific role and relevance of these receptors in physiological function and in pathophysiological states such as hypertension. NPFF receptor based compounds could serve as potential therapeutic agents in the treatment of hypertension and other autonomic disorders.

## Conflict of Interest Statement

The authors declare that the research was conducted in the absence of any commercial or financial relationships that could be construed as a potential conflict of interest.
